# Association of the Degree of Varus Thrust during Gait Assessed by an Inertial Measurement Unit with Patient-Reported Outcome Measures in Knee Osteoarthritis

**DOI:** 10.3390/s23104578

**Published:** 2023-05-09

**Authors:** Shogo Misu, So Tanaka, Jun Miura, Kohei Ishihara, Tsuyoshi Asai, Tomohiko Nishigami

**Affiliations:** 1 Department of Physical Therapy, Faculty of Nursing and Rehabilitation, Konan Women’s University, 6-2-13 Morikita-machi, Higashinada-ku, Kobe 658-0001, Hyogo, Japan; 2 Department of Rehabilitation, Fukuoka Orthopaedic Hospital, 2-10-50 Yanagochi, Minami-ku, Fukuoka 815-0063, Fukuoka, Japan; loose_no_6@ybb.ne.jp (S.T.); nanaki3221@gmail.com (J.M.); 3 Department of Orthopaedic Surgery, Fukuoka Orthopaedic Hospital, 2-10-50 Yanagochi, Minami-ku, Fukuoka 815-0063, Fukuoka, Japan; ishihara@fukuokaseikei.com; 4 Faculty of Rehabilitation, Kansai Medical University, 18-89 Uyamahigashicho, Hirakata 573-1136, Osaka, Japan; asaits@makino.kmu.ac.jp; 5 Department of Physical Therapy, Faculty of Health and Welfare, Prefectural University of Hiroshima, 1-1 Gakuen-tyou, Mihara 723-0053, Hiroshima, Japan; tomon@pu-hiroshima.ac.jp

**Keywords:** gait, varus thrust, patient-reported outcome measures, knee, osteoarthritis, inertial measurement unit

## Abstract

This study aimed to assess the association between the degree of varus thrust (VT) assessed by an inertial measurement unit (IMU) and patient-reported outcome measures (PROMs) in patients with knee osteoarthritis. Seventy patients (mean age: 59.8 ± 8.6 years; women: n = 40) were instructed to walk on a treadmill with an IMU attached to the tibial tuberosity. For the index of VT during walking (VT-index), the swing-speed adjusted root mean square of acceleration in the mediolateral direction was calculated. As the PROMs, the Knee Injury and Osteoarthritis Outcome Score were used. Data on age, sex, body mass index, static alignment, central sensitization, and gait speed were collected as potential confounders. After adjusting for potential confounders, multiple linear regression analysis revealed that the VT-index was significantly associated with the pain score (standardized β = −0.295; *p* = 0.026), symptoms score (standardized β = −0.287; *p* = 0.026), and activities of the daily living score (standardized β = −0.256; *p* = 0.028). Our results indicated that larger VT values during gait are associated with worse PROMs, suggesting that an intervention to reduce VT might be an option for clinicians trying to improve PROMs.

## 1. Introduction

The prevalence of knee osteoarthritis (OA) is increasing worldwide, and it is one of the leading causes of disability and decline in quality of life (QoL) [1,2,3,4]. Patient-reported outcome measures (PROMs) are crucial as they assess not only self-reported symptoms but also disability level and participation status [5,6]. This is important because the patient-reported status is often not correlated with radiographic severity in patients with knee OA [7,8]. The Knee Injury and Osteoarthritis Outcome Score (KOOS) was one of the most commonly used PROMs, having reported psychometric properties, and is a clinically recommended tool [5,6]. Interventions for knee OA should aim to improve PROMs; thus, identifying the factors that influence PROMs is crucial.

Patients with medial knee OA often exhibit an abnormal gait pattern called varus thrust (VT), which is observed in a significant proportion of patients, ranging from 16% to 46% [9,10,11,12,13,14,15,16]. VT is one of the gait abnormalities of the frontal plane in patients with knee OA that can be easily evaluated, which is defined as an excessive “bowing-out” knee motion in the frontal plane during ambulation as the limb accepts weight with a return toward a more neutral alignment in late stance and swing [9]. As walking is the most performed form of physical activity [17,18], the abnormal gait patterns in patients with knee OA could cause unbalanced mechanical stress on the knee joint, worsening of symptoms, and loss of ability. A few studies have examined the association between the degree of VT and PROMs in patients with knee OA [19,20]. Although a study reported that a larger VT assessed using a three-dimensional motion analysis system was associated with worse PROMs [19], other studies reported that no association was observed between the degree of VT and PROMs [20]; thus, the association remains unclear. Clarifying this association would provide clinicians with the impetus to develop effective interventions for PROMs.

Both VT and PROMs have been reported to be affected by many factors, including demographic factors or disease severity. Hence, older age, women, higher body mass index (BMI), and severe Kellgren and Lawrence (K-L) classification were found to be associated with both the presence of VT and worse PROMs [12,13,14,15,16,21,22,23,24,25]. Thus, considering these confounding factors is essential to rigorously examine the association between these two. Additionally, central sensitization (CS) has also received attention recently as a factor that strongly influences PROMs [25,26,27,28]. CS is defined as the increased responsiveness of nociceptive neurons in the central nervous system to normal or subthreshold afferent input by the International Association for the Study of Pain [29]. Many patients with knee OA have CS, which is believed to be due to persistent nociceptive input from an osteoarthritic joint [30]. More recently, a study has revealed that patients with CS showed a gait control pattern different from that of those without it [31]. Thus, CS is also considered an important confounding factor. As a screening instrument, the Central Sensitization Inventory (CSI), which is a self-report measure of CS-related symptoms, was developed to facilitate the assessment of CS in a clinical setting [32,33]; we could use this instrument. Moreover, gait speed is the other important confounding factor because conservative gait patterns, such as slower gait speeds, are assumed to reduce VT, and lower gait speeds are also associated with lower PROMs [21,24]. Nevertheless, no studies have examined the relationship between the degree of VT and PROMs after adjusting for these confounding factors; particularly, no study has included CS and gait speed as confounding factors.

As an objective and quantitative method for assessing the degree of VT, the method using inertial measurement units (IMU) has been reported recently [34,35,36,37,38]. A report demonstrated that the index of VT calculated from acceleration and angular velocity data from an IMU attached to the tibia has good test–retest reliability and good discriminative ability for VT [38]. IMUs are increasingly being used in various clinical settings because of their convenience compared with motion analysis systems [39,40]. However, to the best of our knowledge, no study has examined the association between the degree of VT assessed using an IMU and PROMs in patients with knee OA.

Therefore, this study was designed to examine the association between the degree of VT assessed using an IMU and KOOS (one of the most commonly used PROMs) after adjusting for the potential confounding factors in patients with knee OA.

## 2. Materials and Methods

### 2.1. Subjects

In this study, 70 patients diagnosed with knee OA participated. The subjects were recruited from outpatients scheduled for tibial osteotomy in an orthopedic hospital. The patients with symptomatic medial knee OA diagnosed according to the clinical or radiographical classification criteria of the American College of Rheumatology and with K-L classification ≥ II were included in this study. The exclusion criteria were as follows: (1) patients younger than 40 years; (2) those with lateral knee OA; (3) those with osteonecrosis of the femur or tibia; (4) those with severe pain in areas other than the knee; (5) those with severe neurological diseases, such as stroke or Parkinson’s disease; and (6) those with severe psychiatric diseases, such as depression or dementia, as these diseases could affect the gait pattern or self-reported outcomes.

### 2.2. Assessments of VT during Gait

The protocol to assess the degree of VT was the same as that previously reported [38]. Briefly, the patients walked on a treadmill at a comfortable speed with an IMU attached to the tibia on the affected side. After familiarization with the assessment condition, data measurements were taken at steady, continuous 10-stride walking.

**Figure 1:**
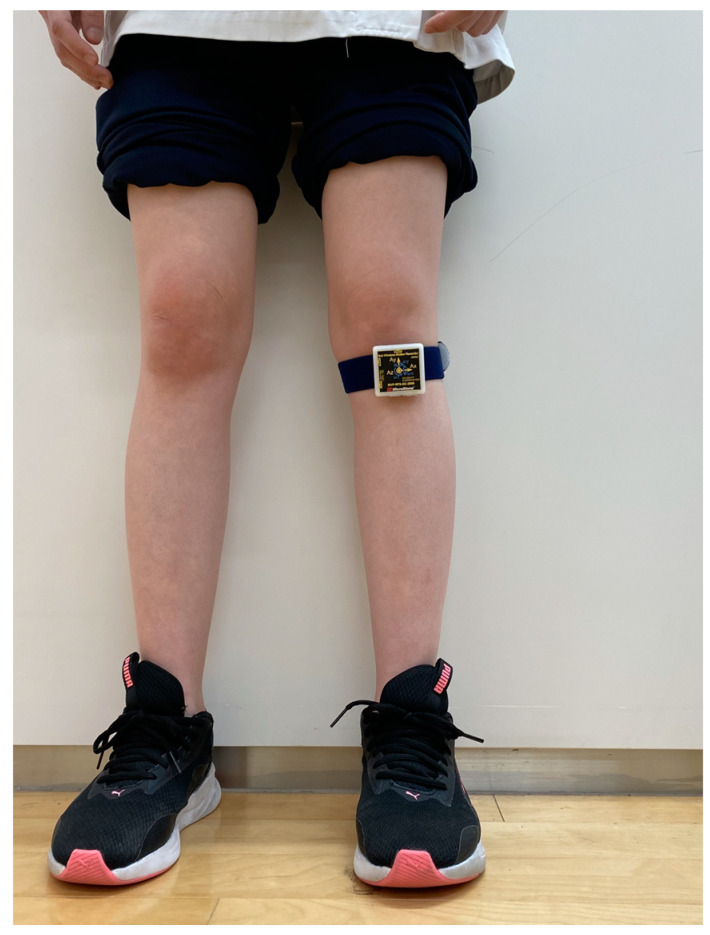
Photograph of the inertial measurement unit attached to the tibia.

The IMU was equipped with a three-axis acceleration sensor (range: ±60 m/s^2^) and a three-axis gyroscope (range: ±2000 degrees/s) (MVP-RF8; MicroStone, Nagano, Japan). The IMU was secured to the tibial tuberosities on the affected side using elastic belts (Figure 1). Acceleration in the anteroposterior, mediolateral (ML), and vertical directions, and angular velocity in the sagittal, frontal, and horizontal planes were measured at a sampling rate of 200 Hz. The obtained data were wirelessly transmitted to a personal computer via Bluetooth.

The obtained acceleration and angular velocity data were analyzed using commercially available software (MATLAB, Release 2019a; MathWorks Japan, Tokyo, Japan). For the index of VT during walking (VT-index), the adjusted root mean square (RMS) of the acceleration at the tibia in the ML direction was calculated. The typical signals of acceleration from the IMU and the calculation method have been described elsewhere [38]. In brief, the VT-index was calculated by dividing the RMS of the acceleration data of the first halves of the stance phase in the ML direction by the mean of the RMS of the angular velocity data of the swing phase in the three planes because the RMS of the acceleration was considered strongly coupled with the swing speed of the lower extremity. A higher VT-index value means a greater degree of VT. The reliability and validity of this index for Japanese populations were confirmed before by the authors [38].

### 2.3. PROMs

As the PROMs, the KOOS for the Japanese population was used in this study [41,42]. The 42-question KOOS consists of 5 subscales: patient-reported pain, symptoms, function in activities of daily living (ADLs), function in sport and recreation (Sport), and knee-related QoL. Each question was scored on a scale of 0–4, with 0 indicating no difficulty and 4 indicating significant knee difficulties. The total score on each subscale is calculated by adding all scores and dividing this value by the mean; high scores indicate no function difficulties. This final score for each subscale ranges from 0 to 100, where 0 represents “severe difficulties” and 100 represents “no problems at all”. This questionnaire is widely used, valid, and responsive to changes in patients with knee OA receiving both nonsurgical and surgical treatments [43].

### 2.4. Potential Confounders

We assessed some potential confounders: demographic data, structural changes and static alignment from radiographical images, and CS. The demographic data of the subjects, including age, sex, height, and weight, were extracted from the medical records. BMI was calculated by dividing the weight by the square of the height. All subjects underwent radiography, and the severity of structural changes was evaluated using the K-L classification. To assess the static alignment of the affected knee, we calculated the Mikulicz line score. The score (%) was calculated by the length from the tibial medial joint surface to the Mikulicz line to the tibial plateau width [44]. Furthermore, we assessed CS using the Japanese version of the CSI [32,33] because CS has been reported to affect both PROMs and motor control strategies during gait [28,31]. The CSI consists of two parts: A and B. Part A is a 25-item self-report questionnaire designed to assess health-related symptoms common in CS syndrome. Each item is rated on a 5-point Likert-type scale (0 = never and 4 = always), with total scores of 0–100. Part B (which is not scored) is designed to determine whether one or more specific disorders, including seven separate CS syndromes, have been previously diagnosed. The Japanese version of the CSI has been validated and shown to have strong psychometric properties [33].

### 2.5. Sample Size

This cross-sectional study set six confounding variables a priori. Hence, seven variables, including the VT-index and the confounding variables, were planned to be included in the multivariate models to examine the association between VT and PROMs. To determine the required sample size for this study, we used two methods. First, according to the traditional method of determining the variables used in the multivariate model (10 subjects per variable) [45], a sample size of 70 was needed. Additionally, we used G*Power, version 3.1, to calculate the sample size for multiple linear regression analysis, with α error of 0.05, power of 0.8, effect size of 0.2, and seven predictor variables. The calculated sample size was 42. As we chose to adopt the larger sample size, we included 70 patients in this study.

### 2.6. Statistical Analysis

All statistical analyses were performed using JMP, version 14.3.0 (SAS Institute Inc., Cary, NC, USA). Data descriptive statistics are presented as means ± standardized deviations (ranges) or numbers and proportions (%), as appropriate. To examine the association between the VT-index and each KOOS score, a single linear regression analysis was first performed. Pearson’s correlation coefficient was also calculated in each examination. Then, multiple linear regression analysis was used to clarify the associations between the VT-index and each KOOS score independently of the potential confounders. Age, sex, BMI, Mikulicz line score, CSI, and gait speed during the assessment of the VT-index were forced to be included in the multiple regression model as confounders. The variance inflation factor (VIF) was calculated to investigate multicollinearity. The level of statistical significance was set at <5%.

## 3. Results

Table 1 presents the characteristics of the subjects. The mean age was 59.8 ± 8.6 years. Of the 70 subjects, 40 (57.1%) were female. The mean BMI was 26.1 ± 3.7 kg/m^2^, and 40 (57.1%) subjects were obese (BMI ≥ 25.0). According to the K-L classification, the number of class II, III, and IV subjects was 19 (27.1%), 45 (64.3%), and 6 (8.6%), respectively. The mean KOOS score ranged from 34.8 for the QoL score to 71.3 for the ADL score. The mean VT-index was 0.036 ± 0.012 m/s/deg (range, 0.016–0.064 m/s/deg). Most subjects (n = 53, 75.7%) exceeded the cutoff value (0.027 m/s/deg) of VT from previous studies [38].

**Figure 2:**
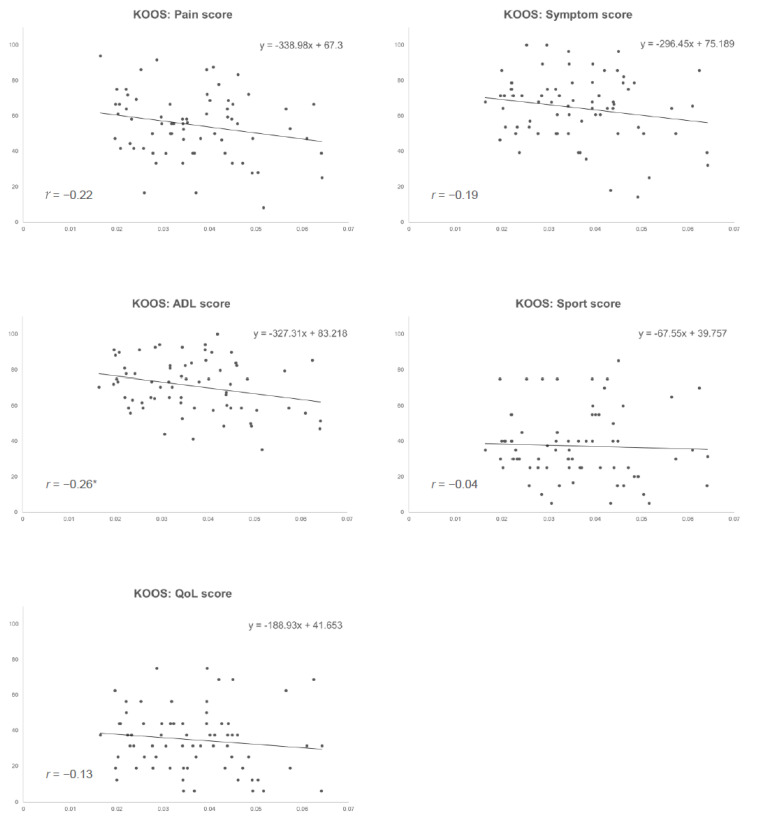
Scatter plots between the VT-index and each KOOS sub-score. The straight lines and equations shown in the figure represent the regression lines and equations in a single linear regression analysis, respectively. Pearson’s correlation coefficients (r) were presented in the lower left corner of each graph. * *p* < 0.05. VT-index, the index of varus thrust during gait; KOOS, Knee Injury and Osteoarthritis Outcome Score; ADL, activity of daily living; QoL, quality of life.

The association between the VT-index and each KOOS score, and the results of the linear regression analyses are shown using scatter plots and regression lines in Figure 2. Pearson’s correlation coefficients were −0.22 (*p* = 0.07), −0.19 (*p* = 0.12), −0.26 (*p* = 0.03), −0.04 (*p* = 0.74), and −0.13 (*p* = 0.30) for the pain, symptoms, ADL, Sport, and QoL scores, respectively.

The results of the multiple regression analyses are shown in Table 2. After adjusting for age, sex, BMI, Mikulicz line score, CSI, and gait speed, the VT-index was significantly associated with the pain (standardized β = −0.295; *p* = 0.026), symptoms (standardized β = −0.287; *p* = 0.026), and ADL (standardized β = −0.256; *p* = 0.028) scores, but not the Sport (standardized β = 0.075; *p* = 0.588) and QoL (standardized β = −0.076; *p* = 0.577) scores. The VIFs of all variables entered into the regression model were <5; thus, there was no multicollinearity.

## 4. Discussion

In this study, we examined the relationship between the degree of VT assessed using an IMU and each KOOS score. The subjects walked on a treadmill, and the VT-index was calculated from the acceleration and angular velocity data measured using the IMU attached to the tibia. As a result, significant associations between the VT-index and the pain, symptoms, and ADL scores were observed after adjusting for potential confounding factors, such as demographic factors, static knee alignment, CS, and gait speed. These results indicate that severer VT was independently associated with worse PROMs (severer pain and symptoms and more disabilities).

Studies have supported our results, showing associations between the degree of VT and PROMs. Some reports have found that patients with VT rated by experts had worse PROM scores [10,14,15,46]. For example, Fukutani et al. performed multivariable regression analyses to adjust for confounding factors and reported that VT rated by experts was independently associated with the Japanese Knee Osteoarthritis Measure score (a type of PROMs) [15]. Additionally, Bensalma et al. found that a larger VT assessed quantitatively using the varus angle during the stance phase measured using a three-dimensional motion analysis system was associated with lower KOOS scores [19]. However, several other reports did not find an association between VT and PROMs [9,12,20]. These conflicting results may be because of the differences in how VT was evaluated (e.g., subjective vs. objective), differences in the severity of knee OA among the study participants, or differences in the PROM questionnaires used. Mahmoudian et al. reported no association between the degree of VT evaluated quantitatively using the varus angle during gait and the KOOS score, possibly because many patients with mild knee OA, which would exhibit a minimal degree of VT (K-L classification: 0 to III, half of them was 0 or I), were included and no analysis was performed to adjust for potential confounding factors [20]. Overall, our study’s results show an association between VT and the KOOS score are plausible, particularly for patients with moderate to severe knee OA.

Among the KOOS scores, significant associations with VT were found for the pain, symptoms, and ADL scores after adjusting for potential confounders, but not for the Sport and QoL scores in this study. The study by Bensalma et al. also showed that larger VT was associated with lower pain, symptoms, and ADL KOOS scores [19]. These results agree with our results, although the Sport and QoL scores were not reported. A recent systematic review and meta-analysis integrating three studies demonstrated that individuals with VT had 3.84 greater odds of reporting pain than those without VT [46], which also supported our results. Mechanical stress on the medial side of the knee joint is considered a factor affecting the disease progression of medial knee OA. The VT during walking was shown to be associated with knee adduction moments [9,47,48]. The repeated mechanical loading on the medial knee joint caused by VT may have exacerbated the pain and other symptoms and may have affected impaired ADLs. However, the impact of abnormal gait patterns on function in sports and recreation and QoL may have been small because these two scores have characteristics different from those of the remaining scores [43].

The univariate analysis for pain and symptom scores revealed that the association did not reach a significant level, despite reaching a significant level in the multiple regression models. As no multicollinearity was found between the variables in the multivariable model, the lack of a significant association in the univariate analysis could be due to confounding effects that weakened the association. Particularly, higher CSI or lower gait speeds may have such a confounding effect. It has been noted that patients with CS have a tight control gait pattern and limited stability [31]. Furthermore, increased CS has been associated with a fear of movement [49]. When people perceive a threat to their walking stability or any form of fear, they exhibit lower gait speed and step length, which is known as a ″conservative gait pattern″ [50]. This gait pattern would decrease the impact on the knee during the stance period, lowering the degree of VT. In contrast, higher CSI or lower gait speeds were associated with worse PROMs [21,24,25,26,27,28]. Therefore, these factors are considered to lower the associations between the degree of VT and PROMs, and the associations were increased after controlling for confounding factors, such as the above mentioned.

Both the VT-index and the CSI were significantly associated with pain, symptom, and ADL scores of KOOS in the multiple regression models. Although it is not intrinsically acceptable to evaluate the effects of CSI on outcomes because it was included in the model as a covariate, the impact of CSI was greater (standardized β: from −0.465 to −0.516) than that of the VT-index (standardized β: from −0.256 to −0.296). These results are reasonable given that CS has been proven to have a considerable influence on PROMs [25,26,27,28]. Nevertheless, the VT-index was independently associated with the scores regardless of CSI, with a moderate level of standardized β. VT is a gait pattern common in patients with knee OA, and imbalanced stress on the knee joint caused by this pattern is considered to aggravate OA symptoms and worsen PROMs. This mechanism is definitely distinct from how CS affects PROMs in patients with knee OA. Thus, we believe that evaluating the degree of VT, in addition to CSI, is important in patients with knee OA.

This was the first study to demonstrate the robust associations between the degree of VT and PROMs after adjusting for potential confounders, to the best of our knowledge. To properly infer associations between clinical data, confounding bias must be removed as much as possible. In this study, the associations between VT and PROMs were examined after the a priori setting of possible confounding factors and the calculation of the sample size. Although discussing the causality is difficult due to the cross-sectional study design, an intervention to reduce VT might be an option for clinicians trying to improve PROMs. For example, several systematic reviews showed that foot orthosis, such as the lateral wedge insole, could reduce the degree of VT and knee adduction moment, which is strongly related to VT [51,52]. Additionally, Huang et al. suggested that gait retraining and the use of knee braces, in addition to the insole, could be another approach to achieve greater improvements in the degree of VT using network meta-analysis of randomized trials [53]. Further studies, such as those with a prospective design or controlled trials, are strongly warranted to confirm the associations reported in this study and the effectiveness of the interventions to improve PROMs for patients with VT.

This study suggests that the VT-index measured using IMUs is a valid parameter for capturing changes in PROMs. As previously demonstrated, the VT-index could discriminate well healthy subjects without VT from patients with knee OA presenting VT, and a cutoff value was determined [38]. In this study, many patients exceeded the VT cutoff from the previous study, indicating a certain range in the degree of VT and further validating the usage of the VT index. The PROM, which was shown to be associated with the degree of VT in this study, is a clinically essential outcome for knee OA, and VT has also been reported to be associated with disease progression [9,16,54]. IMUs are comparatively lower cost, require little preparation for measurement, and are easy to use in clinical settings [39,40]. Therefore, assessing the degree of VT using IMUs would be useful in clinical situations.

Several limitations should be noted in this study. First, the subjects were limited to patients with knee OA scheduled for tibial osteotomy in an orthopedic hospital. Therefore, the disease severity among the subjects was unevenly distributed; particularly, very severe or mild patients were few. Thus, caution should be exercised in generalizing the results of this study. Second, there might be several additional confounding factors that were not measured. To confirm the relationship between VT and PROMs, further validation with a larger sample, including more measurements and patients of varying severities, will be needed.

## 5. Conclusions

This study demonstrated that severer VT assessed using an IMU was independently associated with worse PROMs (severer pain and symptoms and more disabilities) with a moderate level of standardized β after adjustments for potential confounders, such as age, sex, BMI, static knee alignment, CS, and gait speed, in patients with knee OA. The results suggest that assessing the degree of VT quantitatively using IMUs would be useful in clinical situations involving knee OA. An intervention to reduce VT may be an option for clinicians trying to improve PROMs, but further investigations are warranted to fully understand the effects of the intervention.

## Figures and Tables

**Table 1:** Characteristics of the subjects.

Variables		Mean ± SD/n (%)	Range
Age	[years]	59.8 ± 8.6	[41–74]
Sex: Female	[n (%)]	40 (57.1)	
Body weight	[kg]	67.2 ± 11.6	[44.9–94.7]
Height	[m]	1.60 ± 0.09	[1.44–1.82]
BMI	[kg/m^2^]	26.1 ± 3.7	[20.0–38.6]
K-L classification:	[n (%)]		
II		19 (27.1)	
III		45 (64.3)	
IV		6 (8.6)	
Mikulicz line score	[%]	20.3 ± 12.2	[−17.6–39.1]
CSI		9.9 ± 5.2	[1–22]
KOOS score			
Pain score		54.9 ± 18.5	[8.3–93.8]
Symptom score		64.4 ± 18.5	[14.3–100.0]
ADL score		71.3 ± 15.0	[35.3–100.0]
Sport score		37.3 ± 19.7	[5.0–85.0]
QoL score		34.8 ± 17.6	[6.3–75.0]
Gait speed ^a^	[km/h]	1.34 ± 0.36	[0.50–2.50]
VT-index ^b^	[m/s/deg]	0.036 ± 0.012	[0.016–0.064]

^a^ Gait speed represents the comfortable speed during gait on a treadmill for assessing varus thrust. ^b^ The VT-index was calculated by dividing the root mean square of the acceleration data of the first halves of the stance phase in the mediolateral direction by the mean of the root mean square of the angular velocity data of the swing phase in the three planes. SD, standard deviation; BMI, body mass index; K-L classification, Kellgren and Lawrence classification; KOOS, Knee Injury and Osteoarthritis Outcome Score; CSI, Central Sensitization Inventory; ADL, activity of daily living; QoL, quality of life; VT-index, the index of varus thrust.

**Table 2:** Multiple linear regression analysis to examine the association between the VT-index and KOOS scores.

	Dependent Variables										
	Pain Score		Symptom Score		ADL Score		Sport Score		Qol Score		VIF
	Standardized β	*p* Value	Standardized β	*p* Value	Standardized β	*p* Value	Standardized β	*p* Value	Standardized β	*p* Value	
**Independent variables**											
VT-index	**−0.295**	**0.026**	**−0.287**	**0.026**	**−0.256**	**0.028**	0.075	0.588	−0.076	0.577	1.343
**(Covariates)**											
age	0.104	0.407	0.079	0.520	0.046	0.679	−0.085	0.523	0.008	0.955	1.264
sex	0.093	0.458	−0.097	0.429	0.018	0.870	0.209	0.121	0.1	0.449	1.257
BMI	0.084	0.479	0.079	0.495	−0.117	0.265	−0.242	0.057	**−0.347**	**0.007**	1.113
Mikulicz line score	0.061	0.593	0.131	0.241	0.064	0.526	0.017	0.891	−0.025	0.837	1.049
CSI	**−0.465**	**0.001**	**−0.445**	**0.001**	**−0.516**	**<0.001**	−0.250	0.066	−0.238	0.079	1.278
gait speed	−0.054	0.682	−0.006	0.964	0.173	0.138	0.132	0.346	−0.039	0.779	1.371
**adjusted R^2^**	0.246		0.283		0.343		0.048		0.063		

VT-index, the index of varus thrust during gait; KOOS, Knee Injury and Osteoarthritis Outcome Score; VIF, variance inflation factor; BMI, body mass index; CSI, Central Sensitization Inventory; ADL, activity of daily living; QoL, quality of life.

## Data Availability

The data presented in this study are only available to the members of this project. The data will not be shared with others because consent for data sharing was not obtained from the participants.
